# High Speed Terahertz Modulator on the Chip Based on Tunable Terahertz Slot Waveguide

**DOI:** 10.1038/srep40933

**Published:** 2017-01-19

**Authors:** P. K. Singh, S. Sonkusale

**Affiliations:** 1 Nano Lab, Department of Electrical and Computer Engineering, Tufts University, Medford, MA-02155, USA.

## Abstract

This paper presents an on-chip device that can perform gigahertz-rate amplitude modulation and switching of broadband terahertz electromagnetic waves. The operation of the device is based on the interaction of confined THz waves in a novel slot waveguide with an electronically tunable two dimensional electron gas (2DEG) that controls the loss of the THz wave propagating through this waveguide. A prototype device is fabricated which shows THz intensity modulation of 96% at 0.25 THz carrier frequency with low insertion loss and device length as small as 100 microns. The demonstrated modulation cutoff frequency exceeds 14 GHz indicating potential for the high-speed modulation of terahertz waves. The entire device operates at room temperature with low drive voltage (<2 V) and zero DC power consumption. The device architecture has potential for realization of the next generation of on-chip modulators and switches at THz frequencies.

Terahertz (THz) electromagnetic waves are roughly defined over a frequency band of 0.1–10 × 10^12^_ _Hz which corresponds to wavelengths of 0.3–0.003 cm (wavenumber = 3.33–333 cm^−1^)^1^,^2^. This THz band of the electromagnetic spectrum has been traditionally underutilized and is commonly referred as the “THz gap”, where in the past, there has been a dearth of practical components such as high power sources or sensitive detectors^3^,^4^. However, in recent years, tremendous interest has emerged in the research and development of THz devices and related technology (e.g. THz spectroscopy and imaging), fueled by the new availability of high-power sources such as quantum cascade lasers^5^,^6^. Applications of using THz radiation cover broad areas including material identification, imaging, wireless communications, chemical and biological sensing^7^,^8^,^9^,^10^,^11^,^12^.

One of the greatest potentials for THz frequencies could be in the area of high data rate wireless communication where an inherently high carrier frequency of THz wave will support much wider signal bandwidth compared to the radio frequency (RF) bands used today. Wireless communication is achieved by modulating a high frequency carrier wave either in amplitude or phase or both by a modulating signal. Modulators are therefore a key building block in the realization of any communication platform. There have been realizations of THz modulators, especially in THz spectroscopy and imaging systems, where free space THz waves have been modulated using a mechanical chopper, but the modulation speed is limited to few KHz. Efforts have also been made for the realization of high speed THz modulators^13^,^14^,^15^,^16^,^17^,^18^,^19^. However, existing solutions for terahertz modulation are limited to free space propagation of quasi-optical signals^14^,^16^,^17^,^20^, which may be ideal for THz spectroscopy and imaging, but not really suitable for THz communication which requires modulators be integrated with other components such as amplifiers and detectors on a single chip to enable realization of integrated THz transmitter and receiver systems^5^,^6^,^21^. However, there are some challenges in the realization of on-chip terahertz modulator. First, one needs a tunable device with excellent THz electrical properties that can be modulated preferably using electric fields and second, one needs a low loss THz waveguide for interconnect. Finally one needs to be able to integrate this waveguide with the THz tunable device on a single chip for a monolithic implementation. We have realized a highly energy efficient, high performance THz amplitude modulator on a single chip.

The modulator device proposed here is realized from the interaction of confined THz wave within planar metal lines resembling a slot waveguide with a two-dimensional electron gas (2DEG) underneath. In the literature, it is shown that 2DEG supports plasmonic wave propagation, which has been utilized for the demonstration of an ultra-compact interferometer at 10 GHz however, such a device has only been shown to work at low temperatures (4.2 K)^22^,^23^. At room temperature, due to higher carrier scattering in the 2DEG medium, the plasmonic wave propagation is over-damped imposing practical limitations for the realization of THz components using plasmonic approaches^24^. Moreover since 2DEG is physically confined to nanometer thickness, the interaction length of free space terahertz wave propagating normally through the 2DEG is quite small. This results in the low modulation index in case of modulators. For example, it was shown that 2DEG could modulate free space THz wave by only 3% and at with very low modulation speed of up to 10 KHz^25^. A higher speed modulator over 10 MHz was demonstrated using frequency selective metamaterial-based free-space THz modulator at 0.46 THz^14^. The latter demonstrated a relatively high 30% modulation index using metamaterials to couple the incoming THz waves with the 2DEG^14^. While there have been other free space terahertz modulators^16^, a room temperature on-chip version of a high-speed terahertz modulator has yet to be demonstrated. In this paper, we propose such a device with experimental measurements showing gigahertz speed modulation of THz waves for the first time.

## Results

### THz Modulator Device Concept

**Figure 1:**
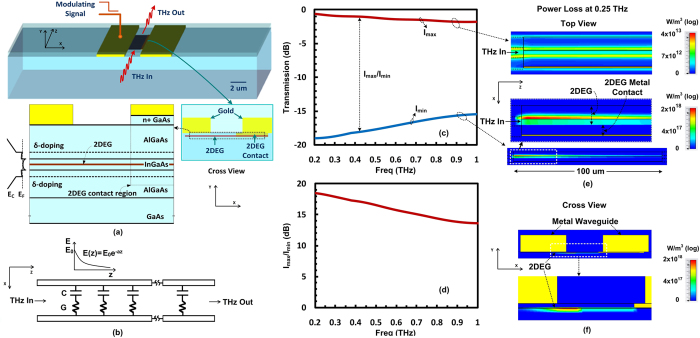
(**a**) Schematics of the THz modulator device, cross-sectional view and active layer structure with contact regions (not to scale) and conduction band energy diagram. (**b**) Equivalent distributed circuit model of device showing capacitor ‘C’ for THz coupling from metal waveguide to 2DEG and 2DEG conductance ‘*G*’, both per unit length (**c**) Device behavior predicted by simulation, simulated power transmission (THz power output/THz power input) for two conditions: power transmission is maximum (Imax) when 2DEG is depleted and minimum (Imin) when 2DEG is present (**d**) The ratio of maximum and minimum transmission, (**e**) Simulated THz power loss density at 0.25 THz (the top view at 2DEG position) for two conditions: first, when 2DEG electron density is minimum or THz transmission is maximum (top figure) and second, when 2DEG electron density is maximum or minimum THz transmission (lower figure), (**f**) Cross sectional view of the simulated THz power loss density when 2DEG electron density is maximum, the most of power loss occurs in 2DEG.

The schematic of proposed THz modulator is shown in Fig. 1(a). It consists a slot waveguide and a 2DEG layer underneath. This slot waveguide is made of two gold metal lines with thickness of 1 μm and width of 2.5 μm separated by a gap of 2 μm. The given dimensions of the waveguide provides wave impedance of 50 ohm to match with the standard impedance of other high-frequency components such as sources and detectors for an effective transfer of power between the device and interconnected components. The increase in thickness of the metal lines of the waveguide and or decrease in the gap between them reduce impedance of the waveguide. The 2DEG lies underneath the metal lines of the waveguide and is in contact with one of them by an additional thin metal layer. The 2DEG extends up to 2 microns underneath the other metal line and is separated from it by an AlGaAs dielectric barrier layer. The 2DEG is formed in InGaAs layer due to the difference in the bandgap between the InGaAs and AlGaAs layers and the transfer of electrons from the delta-doped layers. The electrons in the 2DEG are separated from the ionized donor atoms by the AlGaAs spacer layer resulting in the reduced coulomb scattering and hence increased electron mobility. The InGaAs/AlGaAs/GaAs also forms a pseudomorphic crystal structure with the strained InGaAs layer, which further increases electron mobility. Measured Hall mobility of electrons in this 2DEG is 6500 cm^2^/V.s and carrier density is 3.15 × 10^12^ cm^−2^ at room temperature.

The THz wave propagating in the slot metal waveguide interacts with the 2DEG over its entire distributed length. The finite carrier density of the 2DEG results in a lossy channel for any propagating terahertz wave coupled to it. This results in attenuation of THz waves along the length of the waveguide causing its amplitude to decrease exponentially along the length of the channel as shown schematically in the Fig. 1(b). An equation for the loss in this waveguide is provided in the methods section. Applying a negative bias depletes the electron density in 2DEG reducing its conductance. This in turn lowers the loss experienced by propagating THz waves through this waveguide. Similarly, on the application of positive bias, the electron density in 2DEG increases which results in higher conductance of the 2DEG and higher coupling capacitance thereby increasing the THz transmission loss of the waveguide. In essence, modulating the electron density of the 2DEG causes amplitude modulation of the THz wave propagating through the waveguide. A point to note here is that this function can be easily achieved at room temperature without the need for cryogenic cooling. Also the fact that the carrier concentration in the 2DEG can be tuned electrically makes it attractive for monolithic terahertz integrated circuit implementation.

### Simulation Results

To predict the behavior of the proposed device quantitatively, we have performed a full wave 3D electromagnetic simulation of the device using finite difference time domain (FDTD) simulation using CST microwave studio^®^ software. As mentioned before, in the proposed device the metal waveguide allows low loss propagation of the THz waves and 2DEG underneath works as an electrically tunable loss medium coupled to the waveguide for the purpose of terahertz modulation. The proposed waveguide is analogous to the slot waveguide used at optical frequencies^26^,^27^. This waveguide provides some benefits over other waveguide structures such as coplanar waveguide or plasmonic waveguide^28^,^29^,^30^. Coplanar waveguide requires wider ground metal plane which results in the generation of unwanted leaky surface waves and spurious modes at higher frequencies^28^. The plasmonic waveguide uses single metal line and exhibits high loss for the coupling of THz wave from other devices (source and detector) to the waveguide due to the need for impedance matching^29^,^30^. Moreover, THz wave on such plasmonic waveguide is loosely confined to the metal making it difficult to control THz wave using a finite size electronically tunable medium. As simulation results shows, the proposed slot waveguide provides an excellent confinement of the THz wave energy in the gap between two metal lines of the waveguide (see Supplementary Fig. S1). Moreover the proposed waveguide can also be scaled geometrically to set its wave impedance for matching with sources, detectors and other THz components.

The electrons in the 2DEG are highly confined within an atomic-layer thickness. To incorporate this ultra-thin dimension in the simulation, a 5 nm value for thickness was considered for 2DEG with uniform distribution of electrons. A finite thickness of 2DEG material is required due to the grid-nature of the FDTD simulation. The classical model for conductivity based on Drude’s model works quite well in capturing the dispersion behavior of the 2DEG^14^,^31^,^32^,^33^,^34^. The Drude model for the 2DEG that was used for simulation is provided in the methods section. Note that plasma waves in a medium are heavily damped for frequencies, ω ≪ 1/τ and weakly damped for ω ≫ 1/τ, where ‘τ’ represents the electron scattering time. At room temperature, electron scattering time in the 2DEG is relatively short which results in higher collision frequency, 1/τ of ∼0.72 THz in the presented device. This also implies that plasma waves for frequencies below 0.72 THz will be heavily damped. Traditionally the damping of plasma wave has prevented realization of devices at THz frequencies at room temperature that exploit plasma wave based physics in the 2DEG^20^,^22^. In the proposed device, however, only the loss mechanism emanating from the damping of terahertz wave in the 2DEG is exploited for realization of THz modulator. It could be argued that the atomic-scale thickness of 2DEG does not provide enough interaction to cause sufficient THz modulation^25^. However, since the interaction of the THz wave propagating in the slot waveguide with the 2DEG happens over the entire distributed length of the waveguide, it has a dramatic effect on the THz wave propagation. Moreover, the highly confined gap size in the slot waveguide allows for even greater interaction of the terahertz wave with 2DEG, which is located in and underneath this gap.

Power transmission through the device has been simulated for both low and high electron densities in the 2DEG as shown in Fig. 1(c). The total length of presented device is 200 μm with 100 μm of 2DEG and 50 μm additional waveguide lengths at both sides. In the case of low electron density in 2DEG, power transmission is maximum (I_max_), with some loss (1–2 dB), attributed to losses in the metal and the substrate. This is referred to as insertion loss of the device. As expected, the power transmission loss is high when electron density in 2DEG is increased. In order to compare modulation depth of device, ratio of the maximum to the minimum transmission (Imax/Imin) is plotted in Fig. 1(d). The modulation depth decreases with increase in the frequency. This is because the loss of the waveguide is lower at high frequencies proportional to the decrease in the conductivity of the 2DEG system at high frequencies. The simulation results predict a modulation depth (Imax/Imin) of 18 dB at 0.2 THz and 13 dB at 1 THz for the 2DEG length of 100 μm. A higher modulation depth can be achieved in device with a longer 2DEG length. This performance can be compared with the modulation depth of 3 dB and 20 dB reported in two different free space wave modulators at resonant frequency of 0.4 THz however with considerably high insertion losses^20^,^35^. Also, compared to the ones mentioned, the modulator presented in this manuscript is broadband in nature.

The simulated power loss density in the 2DEG is plotted in Fig. 1(e). The upper part of the Fig. 1(e) shows loss density for a depleted 2DEG, and the lower part of Fig. 1(e) shows loss density for 2DEG with high electron density. Power loss is significantly increased when the 2DEG carrier concentration is high. Also, the power loss decreases exponentially along the length ‘z’ of the waveguide as predicted from Fig. 1(b). Figure 1(f) shows cross-sectional view of the power loss density which shows that most of the THz power is lost in the 2DEG as expected. The maximum power loss occurs where terahertz is coupled from the metal waveguide in to the 2DEG and this loss decreases exponentially along the length ‘x’ of 2DEG. Due to the high damping loss of THz waves in 2DEG, the total decay length is less than 2 μm.

### Measurement Results

**Figure 2:**
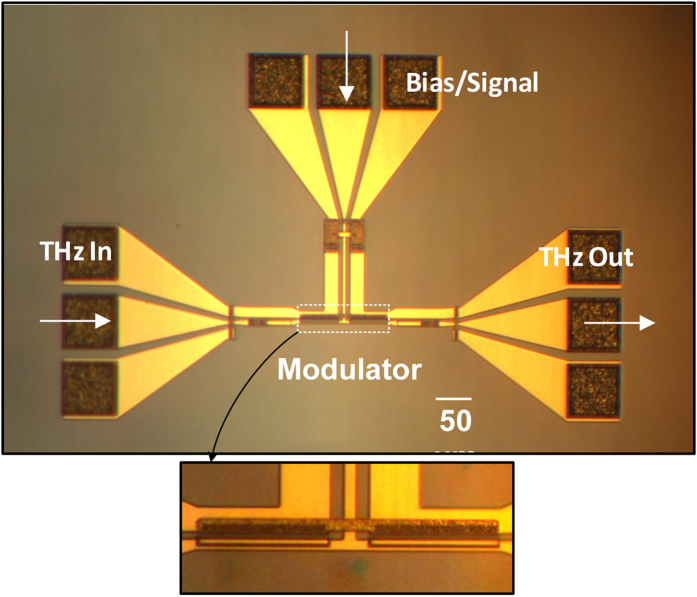
Photo of the fabricated modulator device. The device is designed to operate in the frequency band of 0.22–0.325 THz (scale bar is 50 um). Additional transitions sections are added at input and output of the device to couple THz wave in and out. Another line is added for applying DC bias and modulating signal.

Based on the proposed concept of device and results predicted from the simulation, a prototype device is designed and implemented for the operation in frequency band of 0.22–0.325 THz. This frequency band was chosen solely based on the available experimental facilities for measurement at our laboratory and is not an inherent device limitation. The photo of the fabricated device is shown in Fig. 2. Additional waveguide sections with ground-signal-ground (GSG) pads are added to the modulator device to apply and detect THz waves. Additional waveguide sections are also added to apply DC bias and modulating signal. The additional GSG pad sections are required for the measurement purpose only and will be omitted when modulator is integrated with other THz components on the chip. The simulated power flow through this device is shown in the supplementary section (see Supplementary Fig. S2). Fabrication process for the device is described briefly in the method section.

A customized setup is developed for the measurement of on-chip THz devices (see Supplementary Fig. S3). A free-space continuous wave terahertz generated by heterodyne mixing of two wavelength-offset lasers in a photomixer is used as a source. THz is coupled via a horn-antenna and a ground-signal-ground (GSG) probe to the modulator device. Another set of horn antenna and GSG probe is used to couple THz output wave from the device to the detector. Detector is another photomixer that generates photocurrent proportional to the electric field of the incident terahertz wave.

**Figure 3:**
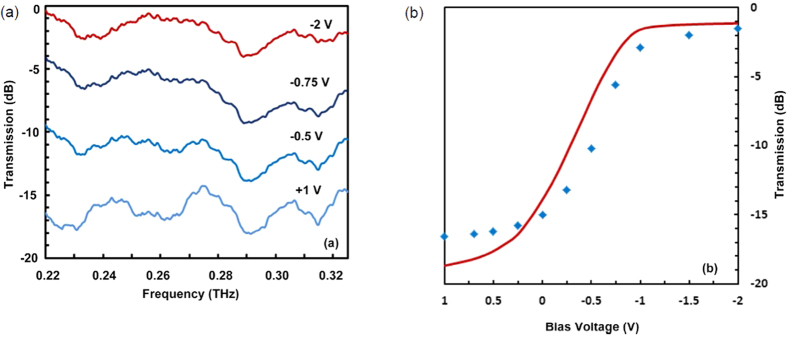
Measured THz transmission behavior of the device. (**a**) Measured power transmission at different DC bias voltages, (**b**) Shows measured (dots) and simulated (continuous line) transmission at 0.25 THz for different DC bias voltages.

For the measurement of transmission through the device, continuous wave THz in the frequency band of 0.220 THz to 0.325 THz is applied to the device (Supplementary Fig. S3(a)). The measured transmissions for different DC biases are presented in Fig. 3(a). At the most negative bias (−2 V), the electron concentration in the 2DEG is mostly depleted, which allows for transmission of THz wave with minimum loss. The higher transmission losses observed at 0.23 THz and 0.29 THz are the effect of additional coupling lines and the bias lines introduced in the device for this measurement. As the amount of negative bias reduces, electron concentration in 2DEG increases which results in an increased transmission loss of THz wave as expected. The transmission loss is at its highest value when the electron concentration reaches its maximum, which happens at the positive bias of +1 V. Also, at this positive bias, higher transmission loss is observed around 0.26 THz, which is attributed to the change in the waveguide impedance at higher 2DEG carrier concentration. The measured transmission results at 0.25 THz for different DC bias voltages is plotted in Fig. 3(b) and also compared with the simulation results. For simulation, electron densities, are estimated from the measured capacitance-voltage characteristics of the device for different DC bias voltages. A close agreement between measurement results and simulation results confirms the validity of the model used in the simulation. It also provides opportunity to conduct accurate design and optimization of the device through modeling and numerical simulations in the future. Also, it should be noted that magnitude of applied DC bias in this device is less than 2 V indicating that device requires very low voltage for its operation.

After characterization of the THz transmission through the device for different DC bias voltages, an experiment was set up to test the modulation speed of the device. In this experiment, a continuous wave THz carrier wave at frequency of 0.25 THz is fed at the THz input port of the device and the spectrum of modulated wave is detected at the output port of the device. The setup for this measurement is shown in Supplementary Fig. S3(b). For modulating signal, a sinusoidal wave is applied at the bias/signal port along with a DC bias voltage of −0.6 V. Since, there is no requirement to provide a DC bias current; the proposed modulator does not consume any static DC power.

**Figure 4:**
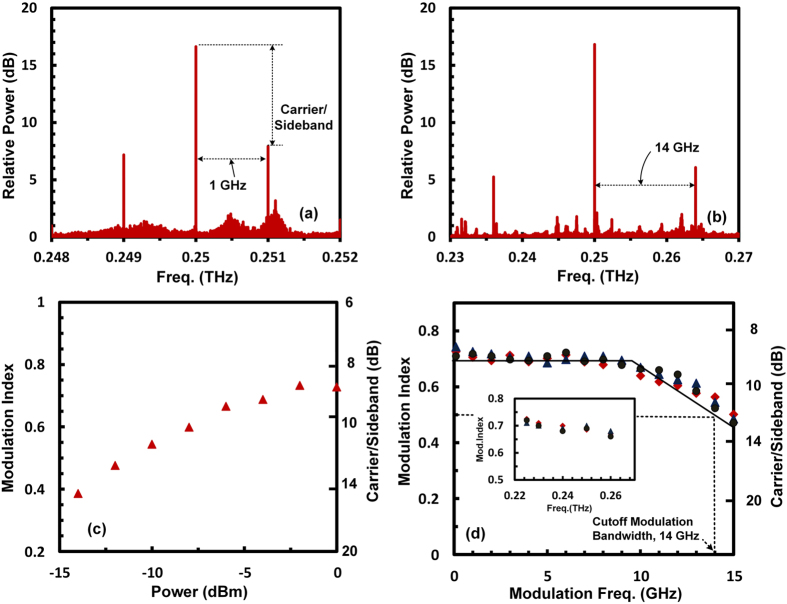
Speed of modulation of the THz modulator. Spectrum of the modulated THz wave, (**a**) Modulation frequency 1 GHz and (**b**) 14 GHz, (**c**) Measured amplitude modulation index with varying input power of modulating signal at carrier frequency of 0.25 THz and modulation frequency of 1 GHz, (**d**) Measured amplitude modulation index at 0.25 THz with different frequencies of modulating signal for 3 number of devices. Inset shows modulation index at different THz frequencies for modulation frequency of 1 GHz.

The measured spectrum of the modulated signal is shown in Fig. 4(a) and (b) for different modulating signal frequencies. Two sidebands and one carrier frequency (0.25 THz) appear in the spectrum of modulated signal, which is a clear indication of the amplitude modulation. The amplitude modulation index is calculated from the power ratio of carrier to the sideband as explained in the method section. Figure 4(c) shows the increase of the modulation index with the increase in the power of the modulating signal. The maximum modulation index of 0.71 is reached at a power level of 0 dBm (1 mW) indicating that this device achieves high modulation index even with very low AC power of the modulating signal. The modulation index for different modulating signal frequencies is plotted in Fig. 4(d). The measured modulation cutoff frequency for the amplitude modulation index of 0.50 (70% of the maximum modulation index of 0.71) is 14 GHz. The modulation cutoff frequency can also be calculated from the charging time (τ_RC_) of the 2DEG capacitor (C, formed between metal and 2DEG), which is charged through the series resistance (R) of 2DEG. The measured capacitance of the device is 1 pF and the estimated series resistance is approximately 10 Ohm, this gives an approximate cutoff frequency (*ω*_C_ = 1/RC) of 15.9 GHz, which is very close to the measured modulation cutoff frequency of 14 GHz. This indicates that modulation cutoff frequency can be increased by decreasing the 2DEG capacitance. This can be achieved by decreasing the coupling length of the 2DEG underneath the slot metal waveguide. However, this will decrease the coupling of the terahertz wave from the slot metal waveguide to the 2DEG, which will ultimately decrease the modulation index. The inset of Fig. 4(d) shows the measured amplitude modulation index for different THz carrier wave frequencies of up to 0.26 THz. The power from THz source decreases with the frequency making it difficult to measure modulation index for carrier frequency above 0.26 THz.

## Discussion

The proposed on-chip THz modulator device is based on the concept of enhanced interaction of THz wave with an electrically tunable two-dimensional electron gas (2DEG) serving as a loss medium. A novel low loss metal slot waveguide is used to confine and couple the THz wave to the 2DEG. Broadband operation for THz modulation is possible using this device. The modulator has been realized and verified experimentally for modulation of electromagnetic waves in the frequency band of 0.22–0.325 THz. This frequency band was chosen based on the available measurement facilities and is by no means a limitation of the device itself. Based on the simulation, one can predict that this modulator can operate over signals up to 1 THz. The modulation frequency exceeds 14 GHz indicating that high-speed modulation of THz can be achieved. The achieved modulation depth is high and can be increased further by increasing the length of the 2DEG in the device at the expense of modulation speed from increase in the charging/discharging time of the 2DEG. The drive voltage required for modulation is really low around 2 V and there is no static DC power consumption. Also, given the fact that this device works at room temperature, it provides a unique advantage, which posits practical applications. All of these properties make the proposed modulator suitable for realization of THz transmitters/receivers on a single chip. Moreover, the high modulation index demonstrated with a short device length implies that one can utilize this device as a high performance on/off switch^16^ at THz frequencies.

## Methods

### Simulation

Electromagnetic simulation was performed to design THz waveguide and predict the interaction of THz wave with the 2DEG. CST microwave studio software was used as a tool for the 3D electromagnetic simulation using FDTD technique.

In the Drude model, the frequency dependent conductivity of the 2DEG is given as


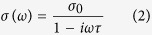


where,


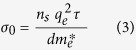


is the DC conductivity. Here, *n*_s_ is sheet carrier density and *d* is thickness of 2DEG, *q*_e_ is charge of an electron (1.6 × 10^−19^ Coulomb), *m*_*e*_^∗^(0.06 × 9.1 × 10^−31^ Kg) is effective mass of electron, and τ is electron scattering time calculated from the electron mobility *μ,* as


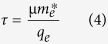


The conductivity of 2DEG is included in the complex permittivity for the electromagnetic simulation and relative permittivity of the material is given as:


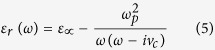


where *ω*_p_ is known as plasma frequency and *v*_c_ is the collision frequency given by;


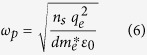


and


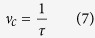


*ε*_*∞*_ = 12.9 (InGaAs) and *ε*_*0*_ = 8.85 × 10^−12^ F/m. The calculated collision frequency is 0.72 THz and the plasma frequency varies with electron concentration.

The amplitude of a wave propagating in a lossy waveguide decreases exponentially as *e*^*−az*^, where *a* is attenuation constant of wave propagating in z-direction. This attenuation constant is proportional to the conductivity introduced by 2DEG to the waveguide and given as 
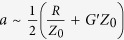
, where *R* is resistance per unit length of waveguide metals, *G′* is effective conductivity per unit length between metal lines mainly introduced by 2DEG, and *Z*_0_ is characteristics impedance of waveguide.

### Fabrication

The fabrication process starts with GaAs wafer (containing active 2DEG layers) and defining active device area. Optical photolithography is used for all processes in defining the microscale patterns. Ohmic contact to the 2DEG is formed by Ge/Au/Ni alloy. A thin layer of GaAs is etched away to reach the barrier layer AlGaAs (separation between metal line and 2DEG) before deposition of the next metal layer. Then metal lines for waveguide are formed by patterning and deposition of Ti/Au layer. Silicon nitride (SiNx) insulator layer is deposited for the formation of metal-insulator-metal (MIM) capacitor. Next, metal layer is patterned to form the top metal of capacitor and bridges. A thick metal layer is deposited at the contact pads. Finally, device is passivated with a SiNx dielectric layer.

### Measurement

Devices are measured using a custom-made setup. The setup involves laser-based generation of a continuous THz wave using a photomixer, coupling of free space THz wave to the device using horn antennas and GSG probes in the band of 0.22–0.235 THz, and detection of the THz wave using a photomixer. The CW THz wave is generated by mixing of two optical waves with different wavelengths in a photomixer semiconductor diode. The detection of THz wave is performed using photoconductive detector. The coupling loss from the probe to device is extracted from the measurements using a reference waveguide. For the measurement of the spectrum of modulated signal a single frequency CW THz source is used as a carrier wave. Modulated signal frequency is down converted using mixer.

The amplitude modulated wave can be presented as:


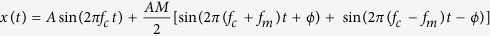


where, carrier wave is presented by *c(t*) = *A* sin(2*πf*_*c*_*t*) and modulating signal by *m(t*) = *M* cos(2*πf*_*m*_*t* + *ϕ*), where M (<1) is the amplitude modulation index. Two sidebands appear at frequencies *f*_c_ + *f*_m_ and *f*_c_ − *f*_m_, and power ratio of carrier to sideband is 4/M^2^.

## Additional Information

**How to cite this article**: Singh, P. K. and Sonkusale, S. High Speed Terahertz Modulator on the Chip Based on Tunable Terahertz Slot Waveguide. *Sci. Rep.* **7**, 40933; doi: 10.1038/srep40933 (2017).

**Publisher's note:** Springer Nature remains neutral with regard to jurisdictional claims in published maps and institutional affiliations.

## Supplementary Material

Supplementary Information
