# Supplementary material for: High Speed Terahertz Modulator on the Chip Based on Tunable Terahertz Slot Waveguide
Source: Sci Rep. 2017 Jan 19;7:40933. doi: 10.1038/srep40933 (PMC5244415; doi:10.1038/srep40933)
Supplement: Supplementary Information [file srep40933-s1.pdf]

High Speed Terahertz Modulator on the Chip Based on Tunable Terahertz Slot Waveguide

Pramod K. Singh and Sameer Sonkusale*

Nano Lab, Department of Electrical and Computer Engineering, Tufts University, Medford, MA-02155.

*** corresponding author email: sameer@ece.tufts.edu**

Simulation: Electromagnetic simulation was performed for the design of on-chip THz waveguide and to understand interaction of confined THz wave in the waveguide with 2DEG. CST microwave studio software was used as a tool for the 3D electromagnetic simulation using FDTD technique. The Drude model is used to consider the frequency dependent conductivity of the 2DEG. The conductivity of the material can be included in the complex permittivity. The relative permittivity of the material is given as:

$\varepsilon_r(\omega) = \varepsilon_\propto - \frac{\omega_p^2}{\omega(\omega - i v_c)} \ldots (1)$; where ω_p is known as plasma frequency and v_c is the collision frequency and given by; $\omega_p = \sqrt{\frac{n_s\ q_e^2}{d\ m_e^*\ \varepsilon_0}} \ldots (2)$ and $v_c = \frac{q_e}{m_e^* \mu_e} \ldots (3)$; n_s is sheet carrier density and d is the thickness of 2DEG, q_e is charge of an electron (1.6×10^{-19} Coulomb), m_e^* ($0.06\times9.1\times10^{-31}$ Kg) and μ_e (6500 $cm^2/V.s$) are effective mass and mobility, respectively for the electrons in the 2DEG at room temperature, ε_∞ =12.9 and ε_0 = 8.85×10^{-12} F/m. The calculated collision frequency is 0.72 THz and plasma frequency varies with electron concentration and maximum value used is 35 THz.

The amplitude of a wave propagating in a lossy waveguide decreases exponentially as e^{-az}, where a is attenuation constant of wave propagating in z-direction. This attenuation constant is proportional to the conductivity introduced by 2DEG to the waveguide and given as $a \sim \frac{1}{2}\left(\frac{R}{Z_0} + GZ_0\right) \ldots (4)$, where R is resistance per unit length of waveguide metals, G is conductivity per unit length between metal lines mainly introduced by 2DEG, and Z_o is characteristics impedance of waveguide.

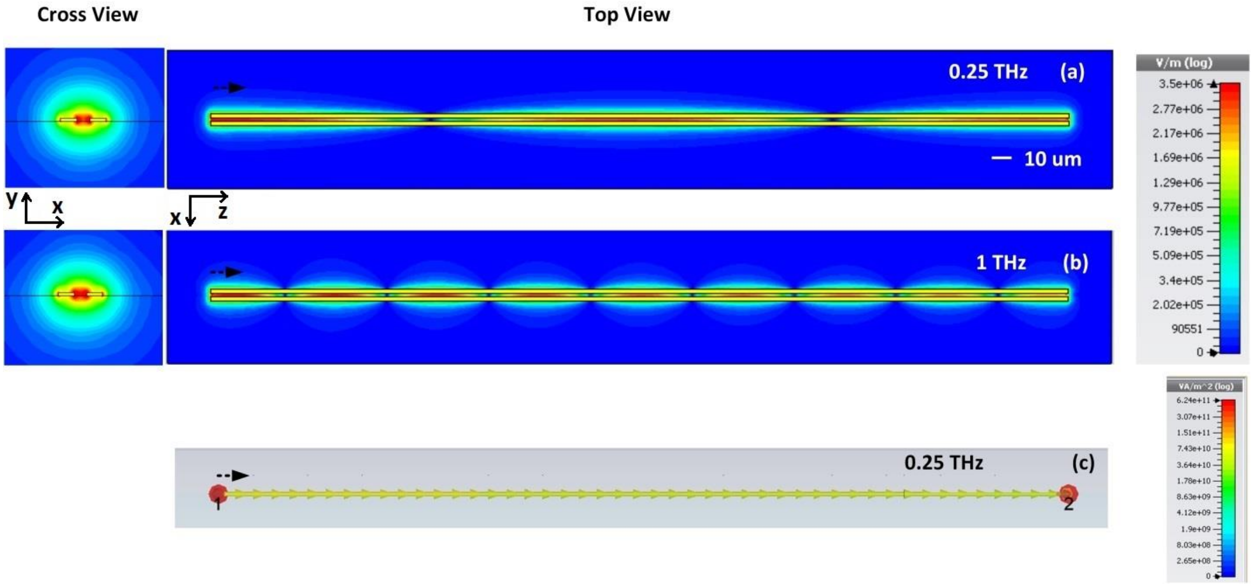

Figure S1. (a) and (b) Simulated electric field distribution in the slot waveguide for the length of 500 µm. (c) Simulated density of power flow in the waveguide. Power is highly confined in the slot.

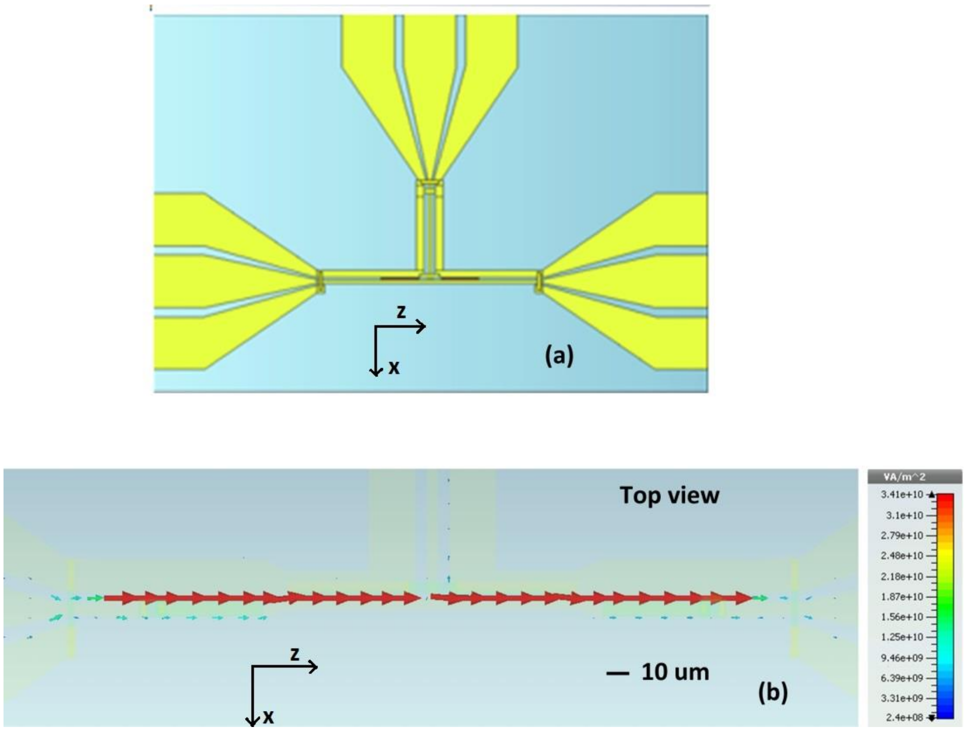

Figure S2. Simulated density of power flow through the modulator device at 0.25 THz.

Measurement Setup: The continuous-wave (cw) THz is generated by optical heterodyne mixing of two different wavelengths of lasers in a photomixer. The THz antenna connected to photomixer emits cw THz wave at the difference (beat) frequency of the lasers. Laser wavelength is in the near IR band (1531.5±3 nm) and photomixer is made on semiconductor (InGaAs). Another photomixer is used for the detection of the THz at receiving end. Frequency of the THz wave is tuned by tuning wavelength of the laser. THz beam emitted from the THz source (Tx) is coupled to the horn antenna (0.22-0.325 THz), which is connected to the waveguide probe for coupling of THz to the input of device under test (DUT), modulator in this case. Second probe and antenna system couples THz wave from output of DUT to THz detector (Rx). THz detector generates photocurrent proportional to electric field of the incidence THz wave, which is further amplified by lock-in amplifier and recorded. The coupling loss from probe to device is extracted from the measurements using a reference waveguide.

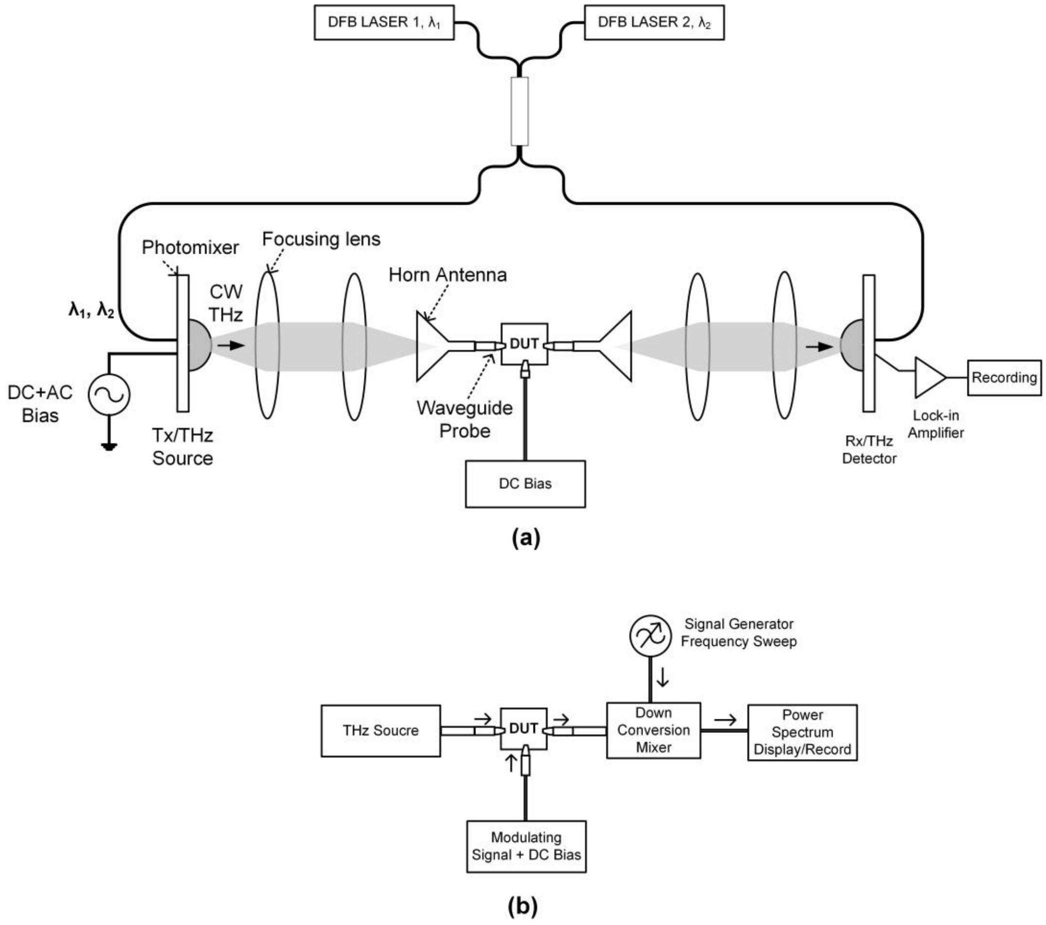

Figure S3. Schematics of the Custom made measurement setup. (a) Broadband transmission measurement at different DC biases. (b) Measurement of modulation speed by measuring spectrum of the modulated signal.

Measurement of the modulation speed requires different setup as shown in the Fig. S3(b). Sinusoidal signal with different frequencies are applied at modulating signal port of the device. Spectrum of the modulated THz wave is recorded after down converting THz frequency using a sub-harmonic mixer. The amplitude modulated wave can be presented as:

$x(t) = A\sin(2\pi f_c t) + \frac{AM}{2}[\sin(2\pi(f_c + f_m)t + \phi) + \sin(2\pi(f_c - f_m)t - \phi)]$...(5)

where carrier wave is presented by $c(t) = A\sin(2\pi f_c t) \ldots (6)$ and modulating signal by $m(t) = M\cos(2\pi f_m t + \phi) \ldots (7)$, M (<1) is the amplitude modulation index. Two sidebands appear at frequencies f_c+f_m and f_c-f_m and power ratio of carrier to sideband is $4/M^2$.
